# Acyltransferases as Tools for Polyketide Synthase Engineering

**DOI:** 10.3390/antibiotics7030062

**Published:** 2018-07-18

**Authors:** Ewa Maria Musiol-Kroll, Wolfgang Wohlleben

**Affiliations:** Interfakultäres Institut für Mikrobiologie und Infektionsmedizin, Eberhard Karls Universität Tübingen, Auf der Morgenstelle 28, 72076 Tübingen, Germany; wolfgang.wohlleben@biotech.uni-tuebingen.de

**Keywords:** natural products, polyketides, polyketide synthases, acyltransferases, engineering, new bioactive compounds

## Abstract

Polyketides belong to the most valuable natural products, including diverse bioactive compounds, such as antibiotics, anticancer drugs, antifungal agents, immunosuppressants and others. Their structures are assembled by polyketide synthases (PKSs). Modular PKSs are composed of modules, which involve sets of domains catalysing the stepwise polyketide biosynthesis. The acyltransferase (AT) domains and their “partners”, the acyl carrier proteins (ACPs), thereby play an essential role. The AT loads the building blocks onto the “substrate acceptor”, the ACP. Thus, the AT dictates which building blocks are incorporated into the polyketide structure. The precursor- and occasionally the ACP-specificity of the ATs differ across the polyketide pathways and therefore, the ATs contribute to the structural diversity within this group of complex natural products. Those features make the AT enzymes one of the most promising tools for manipulation of polyketide assembly lines and generation of new polyketide compounds. However, the AT-based PKS engineering is still not straightforward and thus, rational design of functional PKSs requires detailed understanding of the complex machineries. This review summarizes the attempts of PKS engineering by exploiting the AT attributes for the modification of polyketide structures. The article includes 253 references and covers the most relevant literature published until May 2018.

## 1. Introduction

**Figure 1:**
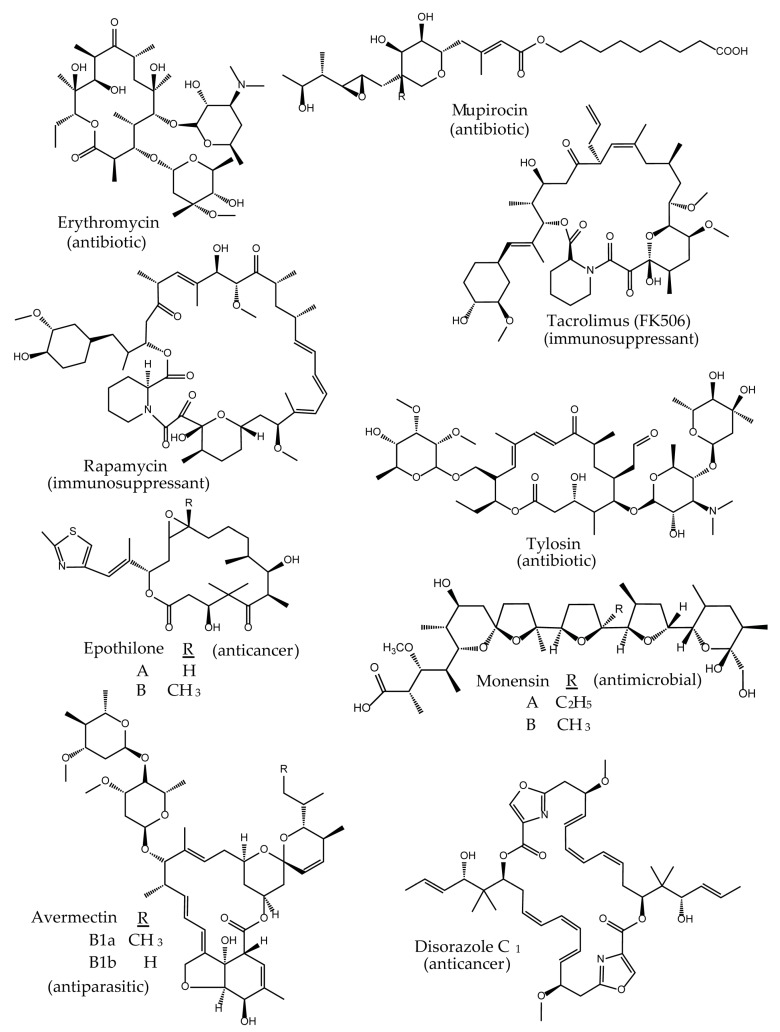
Structures and bioactivity of clinically relevant polyketides.

**Figure 2:**
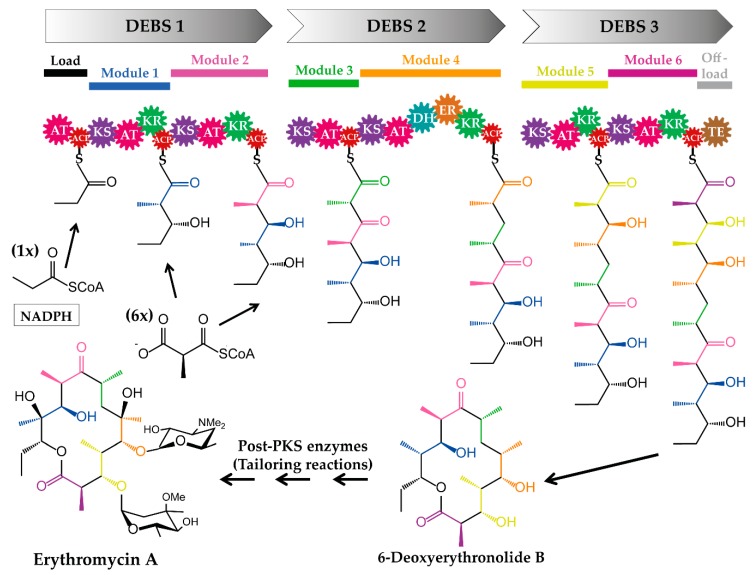
The erythromycin assembly line (DEBS) [20,21]. The prototypical type I modular PKS machinery is composed of three subunits DEBS 1, DEBS 2 and DEBS 3, which are organized into modules. Each module contains a set of domains catalysing one elongation step. The biosynthesis of the chain begins with the loading of the starter unit (derived from propionyl-coenzyme A(-CoA)) onto the loading module (load). Subsequently, the polyketide chain is extended by one extender unit (derived from methylmalonyl-CoA) on each of the “downstream” extension modules. In modules which possess reductive domains (optional), the polyketide intermediate is modified. The synthesized chain is released from the last module (off-load) by the thioesterase domain. Finally, the generated erythromycin intermediate (6-deoxyerythronolide B) is further processed in the post-PKS steps (tailoring). PKS domains: AT, acyltransferase; ACP, acyl carrier protein; KS, ketosynthase; DH, dehydrogenase; KR, ketoreductase; ER, enoylreductase; TE, thioesterase.

Polyketides are a large class of structurally complex compounds with interesting and valuable activities. Those agents are widely used as antibiotics, antifungals, and drugs for other clinical applications [1,2,3]. Erythromycin [4,5], mupirocin [6,7], rapamycin [8,9], FK506 [10,11], and epothilone B [12,13] are examples of antimicrobial, immunosuppressant, and anticancer drugs, respectively (Figure 1). In general, polyketides can be obtained from biological sources (e.g., actinomycetes) or chemically synthesized (semi- or total chemical synthesis). While the isolation of compounds from biological material is rather easy to implement, the chemical synthesis is quite challenging, often limited to certain type of reactions and resulting in low quantities [14]. Therefore, bioactive product discovery methods, which are based on traditional, bioactivity-guided screening and compound isolation are still relevant. There is even a renewed interest in this approach as a strategy for the identification of new drugs [15,16,17,18,19]. In particular, the developments in molecular biology, synthetic biology, sequencing technology, chemistry, and bioinformatics provide new opportunities for the classic screening programs and compound recovery from the natural environment. For example, the combination of the bioactivity-guided screening with sequencing and genome mining approaches supports and accelerates the downstream process of early drug development after a compound has been identified. Furthermore, the recently established methods provide new opportunities, not only for the optimization of the production of low-yield natural products, but they also enable the in vivo/in vitro modification of the molecules, leading to advances in structural diversity. The new perspectives are particularly important for diversification of polyketides, as their structures are complex and thus difficult to access by chemical synthesis. It is the polyketide biosynthesis that offers many opportunities for implementation of the innovative technologies and tools to generate novel analogs. One of such “hotspots” enabling structural modifications of polyketides, are the core assembly lines and their components (Figure 2). 

The polyketide chain synthesizing enzymes, the polyketide synthases (PKSs), often differ in their composition and organization, and the iterative or non-iterative fashion. Based on those features, the PKSs were classified into “different” groups: modular type I PKSs (including the so-called *cis*-AT (AT-containing) type I PKSs and *trans*-AT (AT-less) type I PKSs), iterative type I PKSs, type II PKSs (iterative), and type III PKSs (iterative (chalcone synthase-like PKSs)) [22]. However, in the past two decades many PKS assembly lines were identified, which do not conform to this classification (non-canonical PKSs) [22,23,24,25,26,27]. For example, in aureothin or sceliphrolactam biosynthesis, the modular PKSs AurA [28] and SceQ [29], respectively, harbour an iterative module. Because of such discrepancies, the described classification and terminology are currently used with caution, especially for the newly identified, non-canonical PKS pathways. 

Modular type I PKSs (e.g., the prototypical 6-deoxyerythronolide B synthase (DEBS) from erythromycin) are multifunctional megaenzymes, equipped with domains catalysing the successive linkage of simple building blocks to a polyketide chain (Figure 2). The PKS domains are organized into units termed modules, of which each harbours a set of domains required for one elongation (and modification) cycle. Thus, a minimal PKS consists of the essential domains: the acyltransferase (AT), the acyl carrier protein (ACP) and the ketosynthase (KS). The AT is either embedded into the PKS (*cis*-AT PKS) [30] or encoded as a separate gene, of which the gene product (discrete AT) complements the AT-less PKS (*trans*-AT PKS) [31,32]. In contrast to PKS-independent acyltransferase enzymes, such as dihydroxyacetonephosphate acyltransferases [33] or long-chain-alcohol O-fatty-acyltransferases (wax synthases) [34,35], which typically transfer acyl groups to a non-acyl carrier protein (non-ACP) acceptor molecule, the ATs of polyketide assembly lines load simple units (building blocks) derived from thioester-activated precursors onto the ACPs of a PKS. 

After the ACP-loading step, the KS domain of the PKS catalyses the decarboxylative Claisen-like condensation of the newly loaded unit and the already existing polyketide chain. Additional optional domains, such as the ketoreductase (KR), dehydratase (DH), enoylreductase (ER) and methyltransferase (MT) process the generated β-ketoacyl thioester intermediate. Finally, the polyketide chain is released from the last module of the assembly line, which is usually catalysed by thioesterase (TE). Tailoring enzymes (if present) further modify the intermediate into the final product (Figure 2). 

The structural diversity of polyketides can be assigned to several factors. Those include the length of the polyketide chain, oxidation state and stereochemistry of the β-keto groups, mechanism of termination and chain release, as well as post-PKS tailoring steps [2]. Most of those variations result from the features of the PKS system (type of the PKS, PKS module and domain composition) and modification enzymes. However, the fact that the ATs provide the essential precursors for the polyketide biosynthesis and that they differ in their substrate specificities across the PKS pathways make the ATs to one of the most promising “targets” for engineering of the polyketide assembly lines. Although, the AT-based engineering was successful in some cases, this strategy is still limited by numerous challenges resulting from the complexity of the PKS systems. Therefore, the AT-based engineering is not yet a well-established high-throughput technology. To eliminate or at the very least reduce the engineering bottlenecks and increase the effectiveness of this approach, further knowledge about those complex systems is required. This will contribute to a better understanding of the PKS machineries and enable a more predictable PKS-engineering. 

The goal of this review is to present an overview on the AT-targeted PKS engineering attempts and the recently gained knowledge, which renews the hope for programmable PKS modification and the production of new polyketide derivatives. The synopsis includes an introduction into the PKS assembly lines and its AT-domains, a brief summary of the biosynthesis of polyketide precursors, mechanistic and structural insights into PKSs and finally a more detailed description of the AT-based engineering strategies applied to modular type I PKSs and their future perspectives. 

## 2. Polyketide Synthases and the Essential Acyltransferase Domains

The PKSs are the core enzymes of the polyketide biosynthetic machineries. In modular PKSs, the PKS domains are combined into modules. Each module contains one set of domains required for one elongation and potential modification cycle (Figure 2).

In this review, we discuss the AT-based PKS engineering efforts applied to modular type I PKSs. The structure of a polyketide scaffold, synthesized by a modular PKS assembly line reflects the order of the catalytic domains within the PKS-modules, which usually corresponds to the chromosomal order of the underlying genes. This assembly line-like structure of the textbook modular PKSs (*cis*-AT type I PKSs) and the corresponding products was defined as the “collinearity rule” [21,36,37,38]. It was thereby generally assumed that relying on the sequence encoding the assembly line and the collinear architecture of the modules/domains, including the conserved domain motifs, enable the prediction of the polyketide structure and its stereochemistry [39,40,41]. However, the previous PKS “module” definition, which was deduced from the organization and function of the domains observed in mammalian fatty acid synthase (KS → AT → DH → ER → KR → ACP → TE; from the KS to the ACP) was reconsidered very recently. In 2017, Zhang et al. reported that processing enzymes co-migrate during assembly line evolution with the KS domain downstream (not upstream) of the ACP [42]. Consequently, the term “module”, for modular *cis*-AT PKSs had to be redefined and the polyketide assembly lines updated [43]. According to the newly proposed definition, a PKS module containing all processing domains has the following organization: AT+DH+ER+KR+ACP+KS. Subsequently, 526 ACPs from 33 characterized *trans*-AT PKS assembly lines were analysed using bioinformatic tools [44]. To group the ACPs, “module types” (*a*–*y*) were defined based on the chemistry they perform and classes of enzymes present in the module. A cladogram of ACPs that belong to defined module types was generated and ACP families, which contain related module types, were specified [44]. The analysis uncovered that ACPs from the same module type generally clade together, reflective of the co-evolution of these domains with their cognate enzymes [44,45]. Moreover, cladograms of KSs upstream and downstream of ACPs revealed that in most of the analysed systems, the KSs downstream of ACPs from the same module type also clade together. However, this was not the case for the KSs upstream of ACPs, which was inconsistent with the traditional definition of a module. Therefore, the authors suggested to update the term “module” also for the *trans*-AT assembly lines [44]. We refer to the fact that a new definition of the PKS module was recently introduced. Nonetheless, to avoid any confusion in this review, we rely on the original publications and use the previously reported module nomenclature whenever a PKS assembly line was characterized according to the old module terminology. 

**Figure 3:**
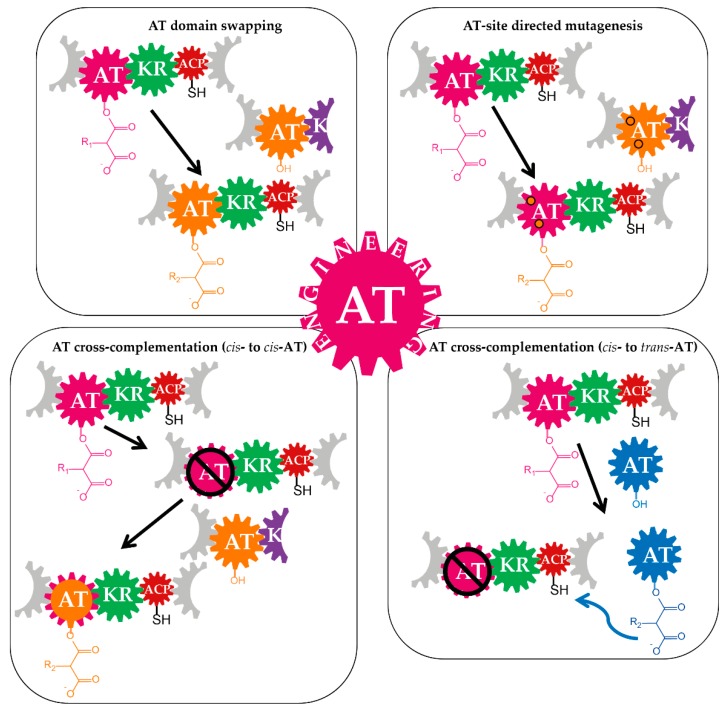
General strategies for acyltransferase-based engineering of polyketide synthases. The acyltransferase (AT)-based engineering of polyketide synthases (PKSs) includes the AT domain swapping, AT-site directed mutagenesis and the AT-cross complementation. In the AT domain swapping approach, the AT (red-purple) of a “target” assembly line is replaced by an AT from another module of the native assembly line or an AT of a foreign pathway (heterologous ATs) (orange). AT-site directed mutagenesis usually involves the identification of conserved motifs, which contribute to substrate specificity of the AT and the mutation of those sites of the AT (red-purple AT with orange circles/black outline). This approach enables the alteration of the AT-substrate specificity. In AT cross-complementation experiments, the AT (red-purple) of a “target” assembly line is inactivated and the PKS module is complemented by a foreign AT (orange or blue). The AT for complementation of the AT-inactivated PKS is either incorporated in place of the native AT (*cis*- to *cis*-AT complementation (orange)) or encoded as separate gene, of which gene product (discrete AT (blue)) complements the assembly line *in trans* (*cis*- to *trans*-AT complementation). Because the outcome of the *cis*- to *cis*-AT complementation is similar to AT-domain substitution or swapping, this strategy is often regarded as such.

The modularity, together with the structural and functional PKS characteristics further strengthened the idea of exploiting the megaenzymes and their domains for diversification of polyketide molecules. The ATs are particularly eligible for these purposes as those domains are responsible for the selection of the precursors and provision of the building blocks (units) to the PKS. More specifically, the precursor- and occasionally the ACP-specificity of the ATs are features that essentially contribute to structural diversity of polyketides and encourage the AT-based PKS engineering. Albeit there are still gaps in understanding of the complex polyketide assembly lines [46,47], various approaches of AT-engineering, including AT domain swapping, AT site-directed mutagenesis, and AT knockout and complementation by an AT-domain from other modules were reported [48,49] (Table 1 and Figure 3). However, it does not necessarily mean that the engineering potential of PKSs by manipulation of the AT features is exhausted. Recent findings such as updates on PKS module boundaries might boost implementation of these strategies and lead to a new breakthrough in this field. Therefore, it seems to be the right time to summarize the available information on the ATs of PKSs, the AT-based engineering of polyketides and the potential new prospects.

### 2.1. Acyltransferase Substrates and Loading of the Acyl Carrier Protein

ATs are indispensable for polyketide assembly as they select the precursors and load the building blocks (units) for the polyketide biosynthesis onto the ACPs of PKSs. However, the ACPs have to be “prepared” for accepting the unit prior to the loading reaction. This is accomplished in a post-translational modification by the 4-phosphopantetheinyl (Ppant) transferase (PPTase) [68,69,70]. The enzyme transfers the Ppant arm of coenzyme A (CoA) to the serine residue of the ACP (*apo*-ACP form), which results in the activated ACP (*holo*-ACP form). The ATs of PKSs load the building block onto the Ppant group of the *holo*-ACP. The units for loading of the *holo*-ACP originate from thioester-activated precursors (e.g., CoA- or ACP-linked units such as acyl starter unit, malonyl units or their 2-substituted derivatives). The loading of the ACP in PKSs is accomplished in a two-step reaction [71]. In the first step, the AT recognizes and binds the precursor to its active serine residues, which results in the acyl-*O*-AT intermediate (self-acylation reaction; step 1). Subsequently, the acyl-*O*-AT interacts with the activated ACP (*holo*-ACP) and transfers the unit onto the protein, which leads to the formation of an acyl-*S*-ACP (transacylation reaction; step 2).

The ATs of *cis*-AT PKSs usually load only one type of extender units (e.g., malonate derived from malonyl-CoA or methylmalonate derived from methylmalonyl-CoA) onto its ACP, which strongly indicates that the ATs are substrate-specific at the native polyketide production conditions. However, there are examples of assembly lines where an AT domain transfers different substrates simultaneously to the same ACP, resulting in the production of polyketide mixtures. This is the case, for example, for the AT domain in module five of the monensin PKS (AT5mon). The monensin AT5 accepts both, ethylmalonyl- and methylmalonyl-CoA as substrates, leading to the production of monensins A and B [72,73] at standard fermentation conditions. The inherent promiscuity of the AT5mon was exploited for loading of synthetic non-natural extender units (allylmalonyl-*N*-acetylcysteamine (allylmalonyl-SNAC), propargylmalonyl-SNAC, propylmalonyl-SNAC, butylmalonyl-SNAC, and hexyl-SNAC) onto the cognate ACP in in vivo feeding experiments [74]. Except 2-hexylmalonyl-SNAC, which was toxic to the producer strain, the synthetic building blocks were incorporated in significant amounts and new premonensin derivatives, including propargyl-premonensin, were produced. This suggests that some ATs might accept a wider range of substrates than originally assumed and that limitations of the availability of non-native precursors for the production of polyketide derivatives can be overcome by feeding with synthetic building blocks. Moreover, it makes the idea of heterologous expression of genes or pathways, directly delivering non-native precursors for the production of polyketide derivatives, very promising (examples for this strain engineering approach were also provided in Section 3.1.1 and Section 3.1.2).

In the following, we briefly summarized the biosynthetic routes for those precursors, which are frequently used by the ATs for the assembly of polyketide structures. 

#### 2.1.1. Provision of Malonyl-CoA and Methylmalonyl-CoA Precursors 

Malonyl-CoA and methylmalonyl-CoA are most commonly used and metabolically available precursors for the biosynthesis of polyketides. In general, there are two pathways for the biosynthesis of malonyl- or methylmalonyl-CoA. One route is the carboxylation of the acetyl- and propionyl-CoA, respectively [75,76]. The acetyl-CoA carboxylases (ACCs) carboxylate acetyl-CoA to malonyl-CoA and propionyl-CoA carboxylases (PCCs) convert propionyl-CoA to methylmalonyl-CoA [77,78]. The other route involves the direct conversion of malonate or methylmalonate to the CoA-activated forms by ATP-dependent malonyl-CoA synthetase and homologous enzymes. Malonyl-CoA synthetases, such as MatB have been described for *Streptomyces coelicolor* [79] and *Rhizobium* [80,81,82]. Hughes and Keatinge-Clay studied the substrate flexibility of the *S. coelicolor* MatB and have shown that this adenylate-forming enzyme is capable of producing most CoA-linked polyketide extender units, as well as pantetheine- and *N*-acetylcysteamine-linked analogs, useful for in vitro PKS studies [79]. It was demonstrated that the methylmalonyl groups ligated by MatB to CoA, pantetheine or *N*-acetylcysteamine were utilized and incorporated into a triketide pyrone by the terminal module of the 6-deoxyerythronolide B synthase (Mod6TE) in vitro [79].

The MatB enzyme of *Rhizobium trifolii* has shown a tolerance for a variety of C2 substituted malonic acids [80] however, in most cases the activity towards the non-native substrates was low. This encouraged the engineering of the malonyl-CoA synthetase and the generation of enzyme mutants, which deliver exotic precursors [65,83,84,85] for polyketide biosynthesis with improved efficiency. 

The incorporation of some of the generated unnatural building blocks into polyketide scaffolds was confirmed in vivo [86,87,88,89,90], which makes the use of the engineered MatB variants attractive for non-chemical polyketide derivatization (“bio-derivatization”). 

#### 2.1.2. Provision of Ethylmalonyl-CoA and Exotic Alkylmalonyl-CoA Precursors

In addition to the typically used substrates malonyl-CoA and methylmalonyl-CoA, some ATs of PKSs utilize unusual alkylmalonyl-CoA precursors for polyketide assembly. Such exotic precursors are generated by crotonyl-CoA carboxylase/reductase (CCR) and homologues (CCRs were broadly defined as enoyl-CoA carboxylase/reductases (ECRs)) [91,92,93,94]. CCRs/ECRs are members of the medium-chain reductase/dehydrogenase (MDR) protein superfamily, which can be found in all three domains of life [95]. The MDR superfamily includes enzymes that reduce either C=O or C=C bonds in α,β-unsaturated carbonyl compounds.

Recent structure-based engineering of the active-site binding pocket of CCRs enabled significant alteration of their catalytic activity towards non-native substrates [96,97]. For example, site-directed mutagenesis of the V350G-site in the CCR enzyme AntE expanded its substrate scope to afford indolylmethylmalonyl-CoA [96]. 

An alternative strategy to the construction of modified biocatalysts for the provision of unusual polyketide precursors is the screening and identification of independent pathways or novel enzymes, directly delivering the exotic substrates. In 2016 Ray et al. described a CCRC (crotonyl-CoA reductase/carboxylase)-independent mechanism for assembly of unusual PKS precursors, where an acyl-CoA carboxylase (YCC, biotin-dependent enzyme) directly carboxylates medium chain acyl-CoA thioesters to alkylmalonyl-CoA [98]. 

The provision of diverse precursors is the prerequisite for the exploitation of the AT promiscuity and the production of polyketide derivatives. Malonyl-CoA synthetases (e.g., MatB-like synthetases) and CCR enzymes enable the generation of a variety of substrates, which can be utilized by the ATs. However, for their use in directed production of AT substrates, the enantiomeric selectivity of the ATs needs to be considered [99]. For example, the AT domains of the model assembly line erythromycin (Figure 2) utilize solely the (*2S*)-isomer of methylmalonyl-CoA for each of the extension step [100]. In such cases the application of CCRCs or biotin-dependent carboxylase enzymes, which generate (*S*)-enantiomers might be the better route to obtain 2-substituted malonyl-CoAs, compared to malonyl-CoA synthetases providing (*R*)-enantiomers. Nevertheless, both enantiomeric forms are useful for testing and characterization of the AT-stereochemistry, which is an important determinant for the efficient incorporation of unnatural moieties into polyketide structures. 

### 2.2. Mechanistic and Structural Insights into Acyltransferases and Polyketide Synthase Modules 

The challenges of polyketide engineering, resulting from the complex modularity and functionality of PKSs became more obvious after some of the initial PKS engineering experiments, including skipping and swapping of domains/modules failed [101,102,103]. Disruptions of the modular architecture of the PKS, destructions of the PKS polypeptide integrity and/or prevention of protein-protein interface interactions were common reasons for the insufficiency of the modified PKSs. This demonstrated that not all paradigms derived from investigations of model assembly lines can be arbitrary transferred to another PKS biosynthetic pathway for its engineering. Accordingly, detailed understanding of these systems is required and indispensable for rational PKS modification. This encouraged the more comprehensive mechanistic and structural analysis of the PKS assembly lines. Here, we focus on AT-substrate and AT-ACP-KS interactions, and present examples underlining the significance of the interdomain communication for the polyketide biosynthesis.

#### 2.2.1. Substrate Recognition and Acyltransferase-Acyl Carrier Protein Interactions

Typically, the ATs accomplish the loading of the ACP by binding of the precursor and the release of the CoASH group prior to the interaction with the respective ACP domain [86,104]. During the loading process, the acyl-AT intermediate is exposed to a nucleophilic attack of the thiol residue of the ACP’s phosphopantetheine moiety. This reaction scenario was termed “ping-pong-bi-bi” mechanism [105]. However, exceptions of this mechanism were observed. For example, the *trans*-AT Dis/Dsz AT from the disorazole pathway exhibited different kinetics, compared to the classical “ping-pong” mechanism. Wong et al. demonstrated that the transacylation in case of the *trans*-AT Dis/Dsz AT depends on the interaction with its ACP before CoASH is released from the building block [106]. 

The significance of the AT-ACP interactions for the transacylation was also confirmed for other assembly lines (e.g., erythromycin, zwittermicin A or vicenistatin). 

In the erythromycin PKS DEBS, protein-protein interactions between AT domains, ACP domains and the linkers that flank AT domains were systematically probed [48,107,108]. The ATs and their cognate ACPs exposed at least 10-fold greater specificity for each other than for heterologous proteins [107]. Moreover, the flanking (N- and C-terminal) linkers of an AT domain contributed to the efficiency and specificity of transacylation, underlining the importance of the linker regions for proper protein-protein interaction [107].

The examples of AT-substrate interactions described above involve ATs which select free precursors and load the building blocks onto ACPs being integrated into the PKS. In addition, AT-substrate recognition and protein-protein “communication” were investigated for the less abundant ACP-linked units (standalone ACPs to which the building block is attached, such as hydroxymalonyl-ACP). In zwittermicin A biosynthesis, the acyltransferase domain of the PKS ZmaA (ZmaA-AT) recognizes the hydroxymalonyl-ACP as substrate and transfers the hydroxymalonyl unit to a downstream ACP via a transacylated AT domain intermediate [109]. The X-ray crystal structure of ZmaA-AT revealed that the ACP itself biases extender unit selection [110]. Furthermore, it indicated that the ACP interaction with the hydrophobic motif promotes secondary structure formation at the binding site and leads to opening of the adjacent substrate pocket lid to allow the binding of the substrate in the active site of the AT [110].

In the biosynthesis of vicenistatin, the AT VinK transfers a very early vicenistatin-intermediate between two ACPs, the standalone ACP VinL and VinP1LdACP (loading module) [111,112]. Thus, it was strongly suggested that the AT VinK needs to distinguish between the ACPs VinL and VinP1LdACP. In a later study, a crystal structure of the AT–ACP (VinK-VinL) complex was obtained, which revealed that Arg153, Met-206, and Arg-299 of VinK interact with the negatively charged helix II region of VinL [113]. The structure of the VinK-VinL complex visualized the interaction between an AT and ACP and provided the first detailed mechanistic insights into ACP recognition by an AT enzyme.

#### 2.2.2. Interactions between the Acyltransferase, Ketosynthase and the Acyl Carrier Protein

The condensation of ACP-bound extender units with the growing polyketide chain is essential for completing the biosynthesis and for the formation of the final product. Those steps (translocation and elongation) are dependent on protein-protein recognition between the AT, KS and the ACP domains [47,114,115,116,117,118]. Regions in the KS domain, KS to AT linkers, and the AT domains of DEBS were described as docking site for the ACP during chain transfer and elongation [118,119]. The protein-substrate and protein-protein interactions cause conformational changes within PKS modules during the catalytic cycle of polyketide biosynthesis. Based on the cryo-EM data obtained for the full-length PikAIII module of pikromycin, Dutta et al. and Whicher et al. described the dynamics of PKS modules as concerted actions to mediate appropriate substrate processing [120,121]. Specifically, the ACP domain is differently positioned after polyketide chain substrate loading onto the active site of the ketosynthase, after extension to the β-keto intermediate, and after β-hydroxy product generation. The conformational rearrangements enable optimal positioning for reductive processing of the polyketide chain elongation intermediate, bound to the ACP [121].

More recently, the synchronized processing of intermediates on a PKS assembly line, was reported as “turnstile mechanism” (chain elongation induces module closure, while chain translocation to the next module reopens the turnstile) [122]. In this study, it was shown that modules, of which the ACP is occupied by an acyl substrate, are not able to load an ACP-borne diketide intermediate onto their KS. The KS was first accepting an acyl chain after the substrate was released from the ACP. However, the exact mechanisms that “communicate” the ACP-loading status to the KS are still unknown. The presumption that the acylation of the KS itself initiates the transfer of the information about the ACP-conformation and ACP-loading state to the KS remains speculative. 

#### 2.2.3. Docking Domains: Intersubunit Communication in Polyketide Assembly Lines

The recent mechanistic and structural data on the PKS assembly lines and their modules provided additional details about these complex systems and uncovered some of the problems, which led to the failure of the initial PKS engineering efforts. From the obtained data, it was possible to generate a more nuanced map of modular *cis*-AT PKS structure and function [49,123], including the determination of so called “docking domains” (DDs) at the N and C termini of the PKSs [124]. The N- and C-terminal DDs ensure the correct PKS assembly into a functional multiprotein complex. For example, the polyketide core of erythromycin A is synthesized by three multienzyme subunits DEBS 1, DEBS 2, and DEBS 3, each harbouring two extension modules (Figure 2). This requires a proper inter-modular transfer between ACP and KS domains as well as inter-protein transfer between the *cis*-AT PKSs. A typical “docking domain”, which enables the intersubunit communication, was identified in the erythromycin assembly line [124] (e.g., between the C terminus of DEBS 2 and N terminus of DEBS 3). The structure of the DD contains two separate four *α*-helix bundles, which together mediate not only specific docking interactions, but also promote dimerization of each homodimer [124]. 

Docking domains were postulated and experimentally confirmed for the less well understood *trans*-AT PKSs [125,126,127]. In this section, we describe the DDs involved in intersubunit communication of the PKS systems, which must not be confounded with the regions, C-terminal to the KS, which were originally proposed as AT-docking domains for the discrete AT in leinamycin biosynthesis (*trans*-AT PKS pathway) [128,129].

Unlike the *cis*-AT PKSs, in which the DDs for intersubunit communication occur between the PKS modules (the docking is C-terminal to the ACP of an upstream polypeptide and N-terminal to the KS of a downstream polypeptide), *trans*-AT PKSs are disconnected within and between modules. Specifically, the junctions between subunits in *trans*-AT PKSs occur within and between PKS-modules [125,126]. Such C- and N-terminal DDs were observed for VirA and VirFG in the *trans*-AT PKS pathway of virginiamycin [125]. In this study, it was shown that the deletion of the C-terminal partner (VirA ^C^DD) or the downstream catalytic domain, significantly affects the N-terminal DD (VirFG ^N^DD). The N-terminal DD (VirFG ^N^DD) exhibited multiple characteristics of an intrinsically disordered protein [125]. The stability of the protein (VirFG ^N^DD) was recovered after fusion of the docking domains. Similar results were provided for the *trans*-AT PKS system of macrolactin [125]. Two-helix, pseudosymmetric motifs of the length of about 25-residue, which non-covalently connect domains between and within the PKS modules were identified. The docking domains and their cognate domain were heterologously expressed and purified as stable complexes from *Escherichia coli* [126], indicating their importance, not only for the correct assembly of the polypeptides, but also for the overall protein stability of the PKS complex. Furthermore, the structure and portability of the four-helix bundle docking domains was demonstrated in deletion and swapping experiments [126]. 

Very recently, a fundamentally different mechanism of intersubunit communication at the KS/DH was reported for the *trans*-AT PKS gladiolin (gbn) [130]. In contrast to the virginiamycin and macrolactin PKSs, which use mutually compatible docking domains at both, the N- and C-termini of the interacting subunits [125,126], the GbnD4 KS domain utilizes a single, largely unstructured docking domain at the C terminus for a direct interaction with the GbnD5 DH domain [130]. The data confirmed that the docking domain is required for the communication of the KS with the ACP, appended to the DH.

Successful engineering of PKS pathways, especially in case of non-canonical assembly lines, requires the identification of crucial interaction regions for the PKS subunits and the elucidation of the mechanism involved in the communication between their domains. The new insights are valuable for the maintenance of proper protein-protein interactions, dynamics, and finally, for the chemical outcome of the modified PKS assembly lines.

## 3. Strategies of Acyltransferase-Based Polyketide Engineering

The most significant and commonly applied strategies of AT-based engineering of PKS pathways are: the AT domain-swapping, site-specific mutagenesis of the ATs and the cross-complementation of an AT-inactivated PKS by another AT, either from the cognate assembly line or using heterologous ATs (Figure 3 and Table 1).

### 3.1. Domain-Swapping

**Figure 4:**
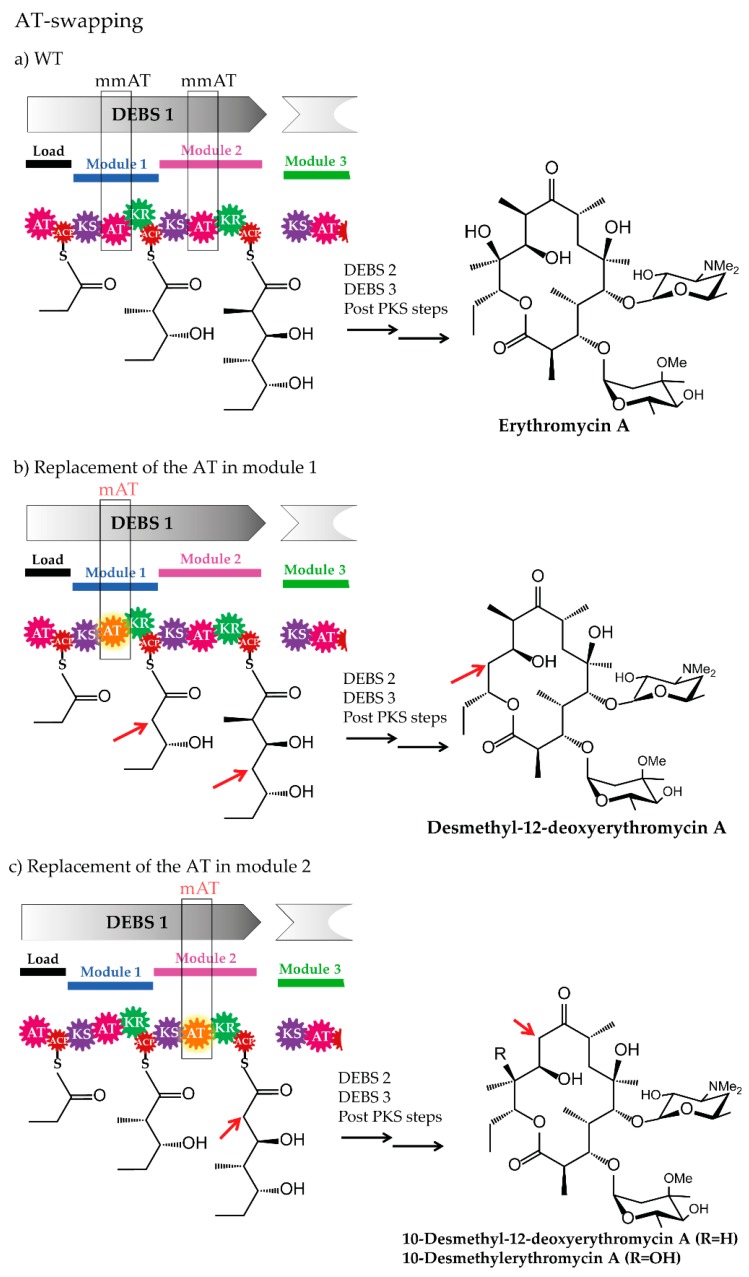
Examples of AT swapping [50]. (**a**) Part of the erythromycin assembly line illustrating DEBS 1 and the final structure of erythromycin A, produced by the wild type strain *Saccharopolyspora erythraea* ER720. (**b**) Mutant in which the AT domain of module 1 was replaced by a malonyl-CoA specific AT (loading malonate onto the ACP). The mutant strain produced the derivative 12-Desmethyl-12-deoxyerythromycin A. (**c**) Mutant in which the AT domain of module 2 was replaced by a malonyl-CoA specific AT (loading malonate onto the ACP). The mutant produced the derivatives 10-desmethyl-12-deoxyerythromycin A and 10-desmethylerythromycin A. Abbreviations: AT, acyltransferase; ACP, acyl carrier protein; KS, ketosynthase; KR, ketoreductase; mmAT: AT loading methylmalonate; mAT: AT loading malonate.

In general, there are two strategies to achieve AT domain swapping in a PKS system. The AT domain can be exchanged by another AT or indirectly replaced by an entire module swapping. For both principles, endogenous ATs/modules from the native assembly line or ATs/modules of a foreign pathway (heterologous parts) can be utilized. In cases where the original AT substrate specificity was altered and functional engineered PKSs were generated, new polyketide derivatives are produced (Figure 4).

#### 3.1.1. Acyltransferase Domain Substitution and the Provision of the Required Non-Native Precursors

One of the most prominent AT-based engineering approaches for PKSs and polyketide structures is the substitution of the AT domain with an AT of a different substrate specificity. The best known example of an assembly line to which this strategy was applied, is erythromycin. In the following, examples of AT-swapping in the erythromycin PKS (DEBS) and in a modified variant of the DEBS (DEBS1-TE), and cases of AT-substitution combined with the optimization of precursor supply are presented. Moreover, the replacement of an AT in a non-erythromycin PKS, is described. 

The erythromycin PKS DEBS was successfully engineered by replacing the methylmalonyl-CoA specific ATs from modules 1 and 2. For example, malonyl-CoA specific ATs from *Streptomyces hygroscopicus* ATCC 29253 (Hyg-AT2 from module 2 of a type I PKS and RAPS-AT14 from module 14 of the rapamycin PKS) and from *Streptomyces venezuelae* ATCC 15439, (Ven-AT from a PKS-like pathway) were inserted into the DEBS of the wild type strain *Saccharopolyspora erythraea* ER720 [50,131]. The mutants of *Sacch. erythraea* ER720, harbouring the engineered DEBS1 produced novel, bioactive erythromycins (12-desmethyl-12-deoxyerythromycin A, 10-desmethylerythromycin A and 10-desmethyl-12-deoxyerythromycin A) [50]. 

The engineering of the DEBS-PKS was also successful in heterologous hosts, such as *S. coelicolor* CH999 [132] and *Streptomyces lividans* K4–114 (in both strains the actinorhodin gene cluster was deleted) [133,134], expressing the entire DEBS-PKS. Liu et al. have shown that swapping of the DEBS-AT from module 6 in the *S. coelicolor* CH999 strain, with the AT originating from module 6 of a rapamycin PKS, leads to the production of the antibacterial erythromycin analog 2-nor-6-deoxyerythronolide B [54]. McDaniel et al. used the two heterologous hosts, *S. coelicolor* CH999 and *S. lividans* K4–114 and applied AT-substitution, combined AT and KR swapping, and other engineering strategies to generate a library of >50 macrolides, including examples of analogs with one, two, and three altered carbon centers of the polyketide products [135].

To minimize the DEBS and achieve a premature release of the polyketide chain from the erythromycin PKS, the assembly line’s TE domain was relocated to the C-terminus of various ACPs (e.g., fusion of the TE to the C-terminus of the DEBS1 resulted in DEBS1-TE) [136,137]. The minimized PKS DEBS1-TE, which consisted of the first two modules of the erythromycin PKS-complex and the TE domain, immediately became a model system for studying not only the mechanistic issues in polyketide biosynthesis [138,139], but also for the engineering of the DEBS-PKS (e.g., AT-domain substitution). Among other experiments, the DEBS1-TE was used to replace the entire AT domain from module 1 by a heterologous AT derived from module 2 of the rapamycin PKS [51]. Two new lactones (2-methyl-3,5-dihydroxy-*n*-hexanoic acid δ-lacton and 2-methyl-3,5-dihydroxy-*n*-heptanoic acid δ-lacton) were produced by *S. coelicolor* containing the modified DEBS1-TE [51]. Although functional engineered PKSs were obtained, the yields of the products generated by these chimeras often greatly depended on the position of the polyketide chain at which the new building block was incorporated and frequently resulted in significantly lower production of the compounds [50,51]. In contrast, Lau et al., generated a variant of the bimodular DEBS1-TE, in which the AT2 domain was replaced with the malonyl-CoA-specific AT2 domain of the rapamycin PKS that produced 10 mg/L of the expected 2-desmethyl triketide lactone [52]. The productivity of the recombinant *S. coelicolor* strain, containing the engineered DEBS1+TE, was 50% relative to the production levels of the parent triketide lactone ((2*R*,3*S*,4*S*,5*R*)-2,4-dimethyl-3,5-dihydroxy-*n*-heptanoic acid δ-lactone), 20 mg/L [52]. 

Further engineering attempts of the DEBS1-TE fusion include the replacement of module 2 of DEBS1-TE with the module 12 from rapamycin or module 3 from the erythromycin assembly line [53,60]. In both cases, engineered PKSs were obtained and triketide lactones, in which for example, acetate extender units were incorporated instead of propionate, were identified in the recombinant *Sacch. erythraea* JC2 strain [140] (*Sacch. erythraea* JC2 is a genome reduced derivative of *Sacch. erythraea* NRRL2338 lacking almost all erythromycin PKS genes). In another study the strain *Sacch. erythraea* JC2 was exploited for the replacement of the AT1 (methylmalonyl-CoA specific) in the model PKS DEBS1-TE with the epothilone EpoAT2 (malonyl-CoA specific in epothilone biosynthesis) and EpoAT3 (utilizes malonyl- and methylmalonyl-CoA at native epothilone production conditions) [55]. Functional PKSs, producing the expected triketide lactone compounds, were generated. However, lower yields of total products were detected when compared to DEBS1-TE (2% and 11.5% respectively) [55]. 

In some cases the alteration of the AT with an AT incorporating a different extender unit is not sufficient as the substrate is not available in the producer and it requires the external supplementation with the precursor or further genetic modification of the strain to overcome this limitation. Such an observation was made for a substitution experiment in which the AT domain of module 4 in erythromycin (eryAT4/methylmalonate-specific) was exchanged with the AT domain from module 5 (nidAT5/ethylmalonate-specific) of the niddamycin PKS [56]. The strain containing the modified PKS produced erythromycin A, but did not synthesize the ethyl-substituted derivative. After supplementation of the culture with ethylmalonate, moderate production of 6-desmethyl-6-ethylerythromycin A (6-ethylEr) was detected in addition to erythromycin A. To eliminate the limitation and improve the intracellular ethylmalonyl-CoA concentration, the crotonyl-CoA reductase (see also Section 2.1.2) from *Streptomyces collinus* [141] was introduced into the *Sacch. erythraea* EAT4, yielding *Sacch. erythraea* EAT4-crr. The *crr*-expressing strain synthesized the derivative 6-ethylErA [56].

Another example of a combined AT-substitution- and precursor supply optimization approach was provided by Kato and co-authors [57]. In their study, the previously designed *S. lividans* K4–114, harbouring a plasmid with genes encoding the erythromycin DEBS was modified: namely, the AT6 domain was replaced by the presumably hydroxymalonate-specifying *fkbA*-AT8 domain [54,58]. This DEBS construction was utilized for a substitution with an AT providing an exotic substrate (methoxymalonate) to the ascomycin assembly line [57]. The *fkbA*-AT8 (originally AT6 in DEBS) of the modified DEBS in *S. lividans* K4–114 was exchanged by the heterologous AT8 domain (loading methoxymalonate onto the PKS) from ascomycin (FK520, synthesized by *S. hygroscopicus*). In addition, a subcluster of five genes (*asm13-17*) from the ansamitocin biosynthetic gene cluster of *Actinosynnema pretiosum* was coexpressed in the modified *S. lividans* K4–114 strain to provide the methoxymalonyl extender unit for the engineered erythromycin PKS. Two novel analogs of erythronolide, 2-desmethyl-2-methoxy-DEB and 2-desmethyl-6-DEB, were produced by this strain [57].

Besides the AT of the erythromycin PKS, other assembly lines were modified by AT-swapping to produce polyketide derivatives. The AT domains in the synthesizing PKS-complex of geldanamycin, a potential anticancer drug, were substituted in six AT swaps (in modules 1, 2, 3, 4, 5, and 7) of the seven GdmPKS modules [59]. The AT-swapping using the RapAT2 domain and/or the RapAT14 domain, both from the rapamycin PKS, resulted in functional PKSs in four (modules 1, 4, 5, and 7) of the six modules. The geldanamycin analogs: 2-desmethyl, 6-desmethoxy, 8-desmethyl, and 14-desmethyl derivatives, including one analog with a four-fold enhanced affinity for its target (chaperone Hsp90, essential for growth of cancer cells), were produced in *S. hygroscopicus* [59].

#### 3.1.2. Examples of Acyltransferase Domain Substitution by Exchanging the Entire Module and the Supply of Non-Native Precursors

The AT substitution resulting from the exchange of entire modules of a PKS assembly line is an indirect strategy and often a “side-effect” of an engineering experiment, primary aiming at alteration of a module and not necessarily at AT-swapping. The attempts of module swapping were recently discussed elsewhere [138,142,143,144,145,146,147]. Therefore, we provide only a few of the most relevant or recent examples for this engineering strategy. 

The exchange of a whole module was successful for loading modules as well as for a chain extension module of a PKS [148,149,150]. For example, the wide-specificity loading module of the avermectin was introduced in place of the first module of DEBS and the resulting hybrid PKS gene was expressed in *Sacch. erythraea* [149]. Novel erythromycins derived from endogenous branched-chain acid starter units were observed, confirming the functionality of the engineered PKS and the flexibility of the assembly line, which accepted and prolonged the polyketide chain with alternative moieties [149].

Replacing of an entire loading module by a loading module of the avermectin PKS from *Streptomyces avermitilis* or the erythromcyin PKS of *Sacch. erythraea* was also successful for the spinosyn PKS in *Saccharopolyspora spinosa* BIOT-1066 [151]. The resulting strain derivatives of *Sacch. spinosa* BIOT-1066, expressing the hybrid PKS pathways produced the anticipated spinosyn analogs [151]. In a previous study, the inherent promiscuity of the loading module of the avermectin PKS was exploited for the generation of a compound library in *S. avermitilis* mutants, blocked in the biosynthesis of native starter units [152]. Structures including the most effective antiparasitic avermectin derivatives (e.g., dormectin) [153] were isolated. More recently, the avermectin biosynthetic pathway was engineered to provide alternative doramectin producers [154]. In this study, the loading module of the avermectin PKS in a *S. avermitilis* strain was replaced with a cyclohexanecarboxylic (CHC) unique loading module from phoslactomycin [154,155]. Furthermore, a CHC-CoA biosynthetic gene cassette was introduced into the engineered strain to ensure the production of the precursor for directed biosynthesis of doramectin. The obtained strain synthesized higher amounts of doramectin (53 mg/L), relative to the yields detected in case of an external supplementation of the wild type strain with the substrate (CHC) (9 mg/L). However, the quantity was significantly lower when compared to the parental avermectin producer (500 mg/L). Nevertheless, the valuable “target” compound (doramectin) could be derived from the module swapping approach and the potential limitations, leading to reduced production yields, might be eliminated by applying diverse strain engineering methods (mutagenesis, synthetic biology etc.). 

In addition to the loading modules, chain extension modules were replaced, as it was the case for DEBS PKS variants. For example, the second module of the bimodular mini-PKS DEBS (loading + DEBS module 1 + module 2) was swapped with cognate modules of the erythromycin assembly line (DEBS module 3 and 6) and heterologous module 5 from rifamycin [150]. The engineered DEBS-PKSs produced the expected triketide lactone in the heterologous expression host *S. coelicolor* CH999 (see also Section 3.1.1). This demonstrates that both approaches (exchanging of whole loading- or chain extension module) can result in engineered PKSs, which are capable of assembling new polyketide derivatives. 

### 3.2. Site-Specific Mutagenesis of Acyltransferases

**Figure 5:**
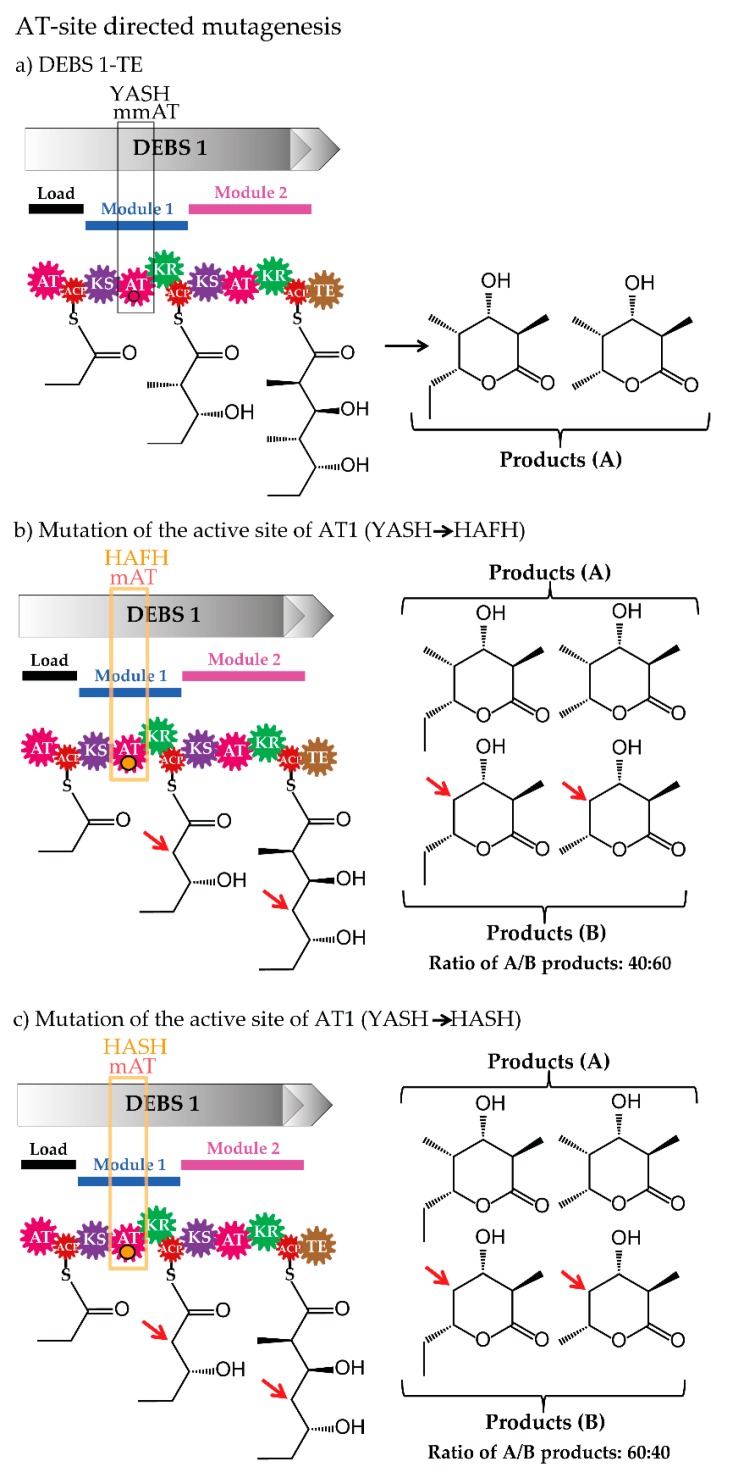
Examples of AT-site directed mutagenesis [60]. (**a**) The fusion of DEBS 1 and TE domain line (DEBS 1-TE) form the erythromycin assembly line. The strain containing the modified fusion DEBS 1-TE produced the respective triketide lactones (products A). (**b**) The fusion of DEBS 1-TE in which the AT of module 1 was modified by exchanging the specificity motif YASH (native, methylmalonyl-CoA) by the HAFH motif (malonyl-CoA). The strain *Saccharopolyspora erythraea* containing the modified DEBS-TE produced triketide lactones A and B (40:60). (**c**) The fusion of DEBS 1-TE in which the AT of module 1 was modified by exchanging the specificity motif YASH by the HASH motif (malonyl-CoA). The strain *Saccharopolyspora erythraea* containing the modified DEBS-TE produced triketide lactones A and B (60:40). Abbreviations: AT, acyltransferase; ACP, acyl carrier protein; KS, ketosynthase; KR, ketoreductase; TE, thioesterase; mmAT: AT loading methylmalonate; mAT: AT loading malonate.

A different approach to the whole domain swapping, aiming at the substitution of AT-substrate specificity, is the site-directed mutagenesis (Figure 5). Recent advances in sequencing methodology, bioinformatics, structural and synthetic biology contributed to the identification of amino acid signatures of ATs and have a significant impact on the engineering of PKS assembly lines [49,61,74,106,113,138,143,144,148,156,157,158,159,160,161,162,163,164,165,166,167,168,169]. 

Analysis of the amino acid sequences of ATs resulted in the identification of approximately 100 residues at the C-terminal end of the active-site serine in the analysed ATs, which was assigned to different AT-substrate specificities. Indeed, in most cases, the substrate specificity of the ATs could be predicted based on this motif. This observation encouraged the modification of the AT substrate specificity by exchanging the specific sequence signature. Such strategy was applied, for example, to the model PKS-complex DEBS and its ATs. The methylmalonyl-CoA specific YASH-motif of AT1, AT4 and AT6 of DEBS was altered to the malonyl-CoA specific HAFH (and HASH) [60,61,62], which resulted in AT variants incorporating both building blocks (methylmalonate and malonate) (Figure 5). Mutagenesis of sequences apart of this motif led to the generation of ATs transferring and loading non-native units onto the PKS however, reduced efficiency was observed [61,62].

Harvey et al. exploited the features of the loading AT by mutating one of the ATs of DEBS and obtaining a new class of alkynyl- and alkenyl-substituted macrolides (e.g., 15-propargyl erythromycin A) with activities comparable to that of the natural product [170]. 

In other studies, the prototypical PKS (DEBS) was subjected to mutagenesis and the generated active site-mutant library was screened for substrate selectivity [61,63,64,65]. Several single amino acid substitutions, having an impact on the selectivity of the PKS, were identified. The substitution Tyr189Arg in DEBS-AT6 inverted the selectivity of the DEBS from its natural substrate toward an alkynyl-modified unit [65]. 

Bravo-Rodriguez et al. used a combined approach, which involved molecular modelling and mutagenesis of the AT6 of DEBS and showed that the V295A mutation of this AT leads to a wider active site and improves the promiscuity for non-native substrates [61,63,64].

Based on AT amino acid sequences, loading ATs (ATs incorporated into the loading module) usually have relaxed substrate specificity and load the loading module with diverse starter units. For example, the loading AT from the modular PKS of avermectin accepts more than 40 carboxylic acids as substrate for the loading module leading to the biosynthesis of a series of congeners [165]. Using modelling tools and site-directed mutagenesis targeting the active site residues, altered specificity toward a panel of synthetic substrate mimics was achieved [165]. 

Very recently, high-resolution X-ray crystal structures of a broadly selective AT SpnD-AT of the splenocin *cis*-AT PKS, accepting long aliphatic chains, up to C7-malonyl-CoA and aromatic benzylmalonyl-CoAs were solved [171]. To the best of the authors’ knowledge, their work provided first structures of an AT-substrate complex and enabled the understanding of both, the stereoselectivity and the broad substrate specificity of the SpnD-AT [171]. Furthermore, using the structural data, it was possible to mutate key residues of the canonical Ery-AT6 from the erythromycin PKS and “shift” its substrate preference. The modified Ery-AT6 was able to accept diverse bulky extender units [171]. These results suggest that future efforts to expand AT substrate tolerance should target the three important residues, Q150, Y278, S280 in methylmalonyl-CoA specific AT or Q150, H278, F280 in malonyl-CoA specific AT [171].

In addition to the AT of *cis*-AT PKS, the discrete AT from the *trans* AT-PKS pathways [31,32,172] was investigated to identify engineering opportunities. While the *cis*-AT PKSs occasionally harbour ATs providing non-malonate building blocks to the assembly line, the ATs of the *trans*-AT PKSs are mostly malonyl-CoA-specific at natural, non-manipulated production conditions. The KirCII-AT [173] from the kirromycin pathway [174] is an exception. This AT loads a branched precursor (ethylmalonate) onto one particular ACP (KirACP5) at native, non-manipulated conditions and therefore belongs to one of the most promiscuous discrete ATs for regiospecific PKS engineering. To map the interaction epitope KirCII:KirACP5, the ACP was subjected to modelling and alanine scanning mutagenesis, where 61 surface residues were individually mutated to Ala [175]. Additionally, several KirCII-mutants were constructed and tested in vitro. The regions involved in the KirCII:KirACP5 interaction, *trans*-acylation activity were identified. Further in vitro investigation of the substrate specificity of KirCII in ACP loading assays revealed that the AT is able to load KirACP5 with allyl- and propargyl-malonate and to a lesser extent with azidoethyl- and phenyl-malonate [176]. Those precursors are not available in the producer cell and the in vivo production of the respective kirromycin derivatives required external supplementation (feeding) with the substrate [90]. Similar in vitro studies were conducted for the discrete AT (malonyl-CoA specific at native condition) from disorazole biosynthesis. Wong et al. used alanine-scanning mutagenesis of one of the ACPs (ACP1 from DSZS) of the discrete AT and identified a conserved Asp45 residue in ACP1, which is co-responsible for the recognition of this ACP as substrate by the AT enzyme [106]. 

While the *cis*-ATs, such as the ATs of the erythromycin assembly line are relatively well studied and were manipulated for several times, there is not much known about the substrate flexibility and the exact mechanisms of partner (precursor or ACP) recognition for ATs from other PKS pathways (e.g., those of the *trans*-AT PKSs). The knowledge obtained from manipulations conducted in the past years will contribute to understanding of the less-explored systems and help to overcome the still existing limitations for AT-based engineering of PKS pathways.

### 3.3. Cross-Acyltransferase Complementation

**Figure 6:**
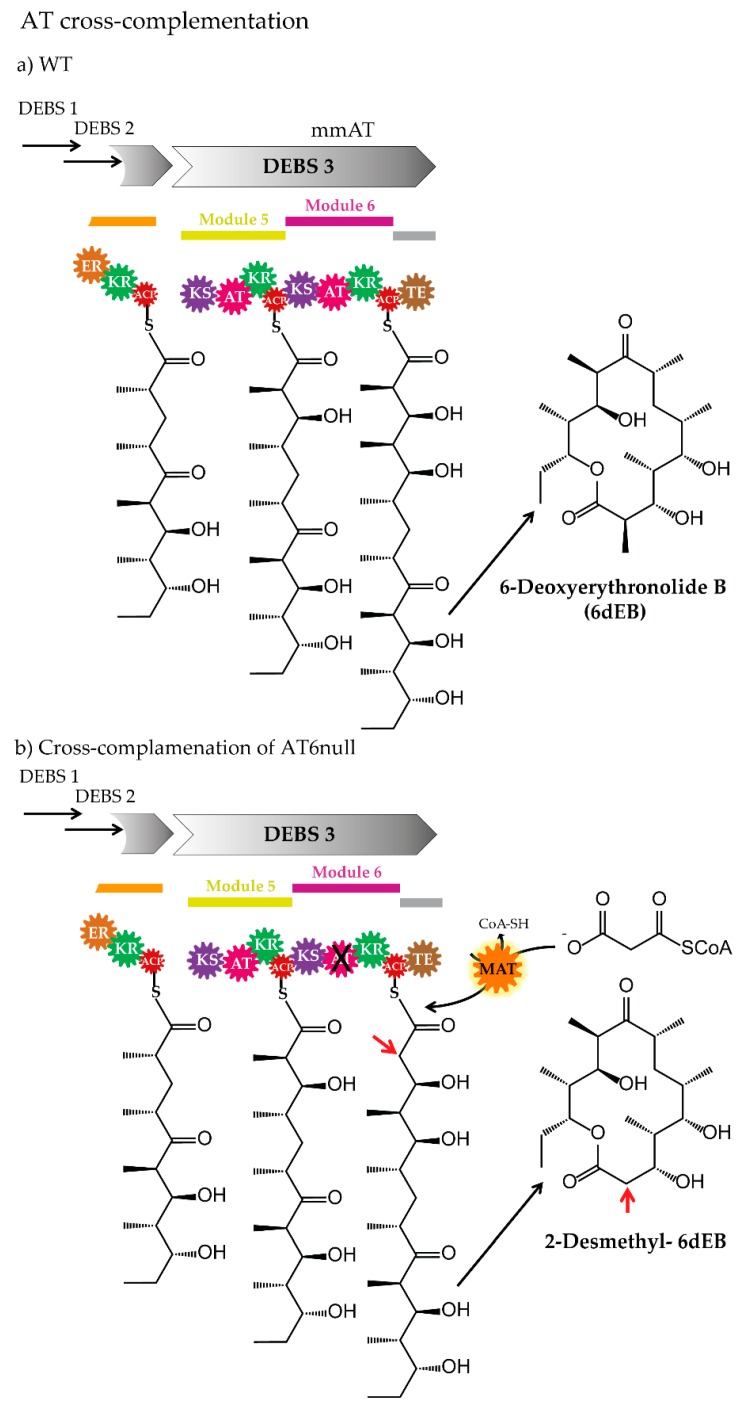
Example of AT cross-complementation [66]. (**a**) Part of the erythromycin biosynthetic pathway illustrating the DEBS complex and the chain intermediates on modules 4, 5 and 6, and the product 6-deoxyerythronolide B (6dEB). (**b**) Part of the erythromycin biosynthetic pathway illustrating the DEBS complex in which the AT6 was inactivated (AT6null) and complemented by a discrete enzyme malonyl-CoA:ACP transacylase (MAT) from *Streptomyces coelicolor*. The strain produced 2-desmethyl-6dEB. Abbreviations: AT, acyltransferase; ACP, acyl carrier protein; KS, ketosynthase; KR, ketoreductase; TE, thioesterase; mmAT: AT loading methylmalonate.

In principle, an AT-inactivated *cis*-AT PKS can be complemented in two ways. One option is the insertion of an AT domain (synthetic, from another module of the native or derived from heterologous assembly lines) in place of the original domain into the PKS module (*cis*-to-*cis* AT-complementation). Another approach is the complementation of the PKS by introduction of a discrete AT, which provides the building blocks in *trans* (*cis*-to-*trans* AT-complementation) (Figure 3 and Figure 6 and Table 1). 

Most of the past AT complementation studies were carried out using the PKSs of the model *cis*-AT PKS assembly line erythromycin from *Sacch. erythraea* and related polyketide pathways. Their modification by deletion of the native AT and integration of a foreign AT, often regarded as domain substitution or swapping, was described in Section 3.1, as well as in diverse reports and reviews [48,49,50,65,138,142,143,144,167,168,177,178,179,180,181,182]. Here, we provide examples of cross-AT complementation, where the functionality of an AT-inactivated PKS (PKS module) was restored by an external heterologous AT protein (e.g., introduction of a heterologous AT into a mutant strain containing an AT-inactivated PKS).

Although the *trans*-AT PKS pathways were overlooked for a long time and are less well characterized than the *cis*-AT PKSs, the number of this type of biosynthetic assembly lines is constantly increasing [32,183]. In most cases, discrete ATs (freestanding ATs or *trans*-ATs) exhibit malonyl-CoA specificity in the native producer strain (e.g., disorazole AT Dis/Dsz [106], bryostatin AT BryP [67], bacillaene AT PksC [184], rhizopodin ATs RizA and RizF [185], and kirromycin AT KirCI [186]) and the diversification of the polyketide chain takes place rather after the elongation by reduction, methylation, and other modifications. However, very few *trans*-AT PKS pathways, which involve discrete ATs with non-malonyl-CoA specificity at native production conditions, were identified. The so far experimentally confirmed examples include the ethylmalonyl-CoA-specific KirCII [173] from kirromycin, the methoxymalonyl-ACP-specific OzmC [187] from oxazolomycins, and the (2*S*)-aminomalonyl-ACP-specific ZmaF [109] required for zwittermicin A biosynthesis. The discrete ATs might exhibit relaxed substrate specificity when the respective precursor is available. This was shown for example for KirCII [90,173,176]. Considering this observation and the fact that the freestanding ATs are encoded by separate genes and act *in trans* to complement the cognate PKSs, their development to a tool for PKS engineering might be easier, compared to the in the ATs embedded in the PKS (*cis*-AT PKSs). In in vitro studies, it was demonstrated that a *trans*-AT from kirromycin (KirCII) and disorazole (Dis/Dsz AT) can complement the AT-null DEBS of the *cis*-AT type [172]. In the past, the AT-null-DEBS module 6 (DEBS, in which the AT domain of module 6 was deleted) was used for similar complementation experiments. For instance, the functionality of the AT-inactivated module was restored after the supplementation with malonyl-CoA:ACP transacylase from *S. coelicolor* (Figure 6), which led to the production of 2-desmethyl-6-dEB [66]. The complementation of AT-null DEBS module 6 was also demonstrated in combination with the AT-domains of the discrete tandem AT BryP from the bryostatin PKS [67] (from ca. *Endobugula sertula*- bacterial symbiont of the marine bryozoan *Bugula neritina*). BryP was also able to catalyse the acyl-transfer onto ACPs of pikromycin and other bryostatin PKS modules [67]. 

In addition, an in vivo cross-species complementation, using ATs from two *trans*-AT PKSs was successful for the ∆*mmpC*-AT1 mutant from mupirocin [188] (*Pseudomonas fluorescens*). *P. fluorescens* produces a mixture of several pseudomonic acids, named mupirocin [189]. Constructs for BryP-AT1 and the didomain protein BryP-AT1AT2 complementation were introduced into the ∆*mmpC*-AT1 mutant [67]. The complementation of the ∆*mmpC*-AT1 mutant expressing the BryP-AT1 restored the biosynthesis of the main compound of mupirocin, the pseudomonic acid A (PA-A), to approximately 83% of WT production levels. 

The efficiency of *trans*-AT complementation is often affected by the identity of the building block and the ACP [172,175]. Thus, the successful complementation and production of efficient modified PKSs still needs a better understanding of the discrete AT-substrate (AT-precursor and AT-ACP) recognition and interaction as well as experimentally verified data, which disclose if the gained engineering knowledge can be transferred to diverse assembly lines or which modifications are required to recover the systems.

## 4. Advances in Natural Science and Future Perspectives of AT-Based PKS Engineering

The recent progress and advances related to polyketides and their biosynthetic machineries provide valuable and essential know-how, which makes the AT-based engineering of PKS assembly lines even more promising than it was before. Nonetheless, this strategy is not yet a standard, high-throughput technology, which is applicable to any desired PKS enzyme. To enable an extensive and efficient construction of functional polyketide machineries, further analysis and experimentally confirmed insights into the complex systems, including non-canonical PKSs are indispensable. The understanding of the structural, mechanistic, catalytic, biochemical details not only for individual domains, but the whole assembly line would make the engineering and tuning more predictable and executable. Furthermore, directed optimization of the producer cell “hosting” the biosynthetic complex would lead to improvements in the productivity of those engineered systems. Today’s sequencing technologies, bioinformatics, structural biology, chemistry, molecular biology, and other in vitro and in vivo tools facilitate the further investigation of the PKS systems and the PKS engineering. Researchers aiming at AT-based PKS engineering will most probably continue to use the established and approved strategies and methodology. Those include in general, the expanding of the pool of precursors [97,98,190] for the ATs, the modification of the AT enzymes under the consideration of the obtained structural and mechanistic insights, AT-domain swapping and cross-complementation of inactivated ATs with ATs from other modules/pathways, and site-directed mutagenesis to exchange the substrate specificity of the AT. Specifically, the information existing in databases [191,192] is incredibly helpful, not only for direct sequence alignments and phylogenetic analysis [193], but also for the development of advanced bioinformatics tools supporting the AT-targeted PKS engineering [194]. The application of such tools allows the identification of new PKS pathways [195] and/or novel types of ATs, the prediction of AT substrate specificity [61,62,158], the analysis of amino acid coevolution in protein sequences [196,197,198], and even protein interaction surfaces [41,199,200]. Recently, the computational online platform ClusterCAD, which facilitates the selection of natural *cis*-AT PKS parts to design novel chimeric PKSs for the biosynthesis of small PKS-derived compounds, was developed [144,201].

The protein structures of PKS domains [112,127,162,169,202,203,204,205], including the ATs [113,161,162,171,184] and whole modules [120,206] enable a more reliable modelling of the architecture of those enzymes and will improve the structure-based PKS engineering. Diverse protein modelling platforms are already available [207,208,209,210,211,212] and were successfully exploited. For example, the modelling of the erythromycin DEBS AT6 with (2*S*)-methylmalonyl-CoA confirmed the role of the proposed active-site residues and revealed residues important for the substrate binding [61]. The obtained information was used for simulations on mutants with the native methylmalonyl-CoA and non-native malonyl-CoA extender units, which led to the identification of residues prohibiting the binding of the desired substrate. Consequently, the respective sites were mutated and mutants, which were able to utilize the substrate 2-propargylmalonyl-SNAC were generated [61].

In another study, the small-angle X-ray scattering (SAXS) was used to characterize the structure of a module (*apo* module 5 from the VirA subunit) and to identify the positions of domains flanking the ACP and KS in the in the virginamycin PKS [213]. The outcome of this analysis enabled modelling of the complete intersubunit interface in the virginiamycin *trans*-AT PKS system [125]. 

Those results are encouraging the implementation of the computational tools, which contribute to the better understanding and more efficient engineering of the complex PKS assembly lines. It is very likely that the bioinformatics software tools will become more important due to their intensive use for diverse simulations and analysis of structural details of PKSs and their AT domains, as well as for the design of engineering experiments in the future. Furthermore, the existing databases will be complemented with the knowledge (e.g., structural data on non-canonical ATs) gained from the planned and currently conducted research on PKSs. This will lead to the development of new computational tools (or tool features) and improve the predictability of PKS-variation as well.

In addition, the structural biology technique itself is constantly improving. A new method for the direct delivery of the sample into an X-ray free-electron laser was used for the *trans*-AT from the disorazole PKS [161]. The novel sample extractor efficiently delivered limited quantities of microcrystals directly from the native crystallization solution into the X-ray beam at room temperature. A crystal structure of the discrete AT with resolution of 2.5 Å was obtained [161]. The crystallization of this difficult to handle enzymes and their complexes (e.g., AT-substrate) is highly desired, as it will provide useful information about the ATs and their interaction partners. We speculate that the progress in this field will continue and support the engineering efforts. 

Directed evolution [214,215,216,217,218] was successfully applied to generate enzyme libraries. This approach usually uses saturation mutagenesis, error-prone polymerase chain reaction (PCR) or DNA shuffling to generate a library of mutated proteins. Subsequently the protein variants are screened in an adequate assay to identify the most promising mutants. The selected mutants are subjected to another cycle of mutagenesis to “evolve” the enzyme to the favoured protein version. This artificial process of repeating cycles of mutagenesis and selection mimics the evolution in the Nature. Although the high throughput directed evolution of PKSs and the AT enzymes is mainly limited by rapid screening (assays and analytics for detection of the of the enzyme catalysis), progress can be observed [219,220,221]. The directed evolution screening/selection strategies, which have been employed to improve or modify the functions of nonribosomal peptide synthetases (NRPSs) and PKSs were summarized in a review [222] recently published by Rui and Zhang. Directed evolution strategies enable the identification of residues affecting the enzyme properties, which might be difficult to detect using alternative methods. Thus, the directed evolution approach provides a good alternative for PKS and AT optimization. 

In the future, those AT-based PKS engineering strategies and methodologies, which rely on DNA steps, will be supported by advanced cloning methods such as Red/ET system [223,224,225], the TAR cloning [226,227], USER cloning [228,229,230], CRISPR-Cas9 [231,232,233,234,235], and other synthetic biology tools [138,143,144,236,237,238]. 

Finally, the efficient production of natural and engineered polyketides often requires the tuning of the native producer strain or the development of suitable heterologous expression hosts [182,239,240]. Preferably, diverse actinomycetes [241,242,243,244,245], *E. coli* [246,247,248], *Bacillus* [249], *Saccharomyces* [250,251] and/or *Aspergillus* strains [252,253] were used for heterologous expression of polyketide pathways in the past. It is very likely, that the existing hosts will be further optimized and specifically adapted to the expression of engineered pathways, including AT-modified PKSs. Most probably, new potential hosts will be identified for the needs of expression of the respective assembly lines and the biosynthesis of the final engineered compound. 

## 5. Conclusions

In the past, many attempts of combinatorial biosynthesis, aiming at engineered PKSs resulted in inactive or inefficient enzymes and/or assembly lines. The newly obtained knowledge about the ATs and their PKS pathways in combination with methodology advancements provide exciting new perspectives for the AT-based PKS engineering. The design and generation of functional and efficient PKS pathways might enable the production of new bioactive compounds of which structures would be extremely difficult to access using traditional synthetic chemistry approaches. 

## Figures and Tables

**Table 1:** Examples of acyltransferase-based polyketide synthase engineering. Abbreviations: acyltransferase (AT); methylmalonyl-coenzyme A (MM-CoA); malonyl-CoA (M-CoA), methoxymalonyl-ACP (MeO-ACP).

Engineering Strategy	Examples			
	Acceptor PKS (Target Assembly Line for the Engineering)	Tool (e.g., Donor AT or Motif) and Origin (Pathway)	Substrate Specificities Native AT (Acceptor PKS)/Used Tool (AT or Motif)	References
AT-swapping	AT from module 1, and 2/erythromycin	“Hyg” AT2 (module 2)/“Hyg” PKS gene, AT (module 14)/rapamycin, Ven AT/unknown PKS-like gene cluster	MM-CoA/M-CoA	[50]
	AT from module 1, and 2/erythromycin	AT (module 2)/rapamycin	MM-CoA/M-CoA	[51,52]
	AT from module 1/erythromycin	AT (module 3)/erythromycin	MM-CoA/MM-CoA	[53]
	AT from module 6/erythromycin	AT (module 6)/rapamycin	MM-CoA/M-CoA	[54]
	AT from module 1/erythromycin	AT (module 2)/epothilone	MM-CoA/M-CoA	[55]
	AT from module 1/erythromycin	AT (module 3)/epothilone	MM-CoA/promiscuous MM, M-CoA	[55]
	AT from module 4/erythromycin	AT (module 5)/niddamycin	MM-CoA/EM-CoA	[56]
	AT from module 6/erythromycin	hydroxymalonate-specifying *fkbA*-AT8/ascomycin (FK520)	MM-CoA/Methoxymalonyl-ACP	[57,58]
	AT from module 1-5 and 7/geldanamycin	AT (module 2 and 14)/rapamycin	MM-CoA, MeO-ACP/M-CoA	[59]
AT-site directed mutagenesis	AT from module 1 and 4/erythromycin	consensus motifs (YASH/HAFH motif)	MM-CoA/M-CoA	[60,61,62]
	AT from module 6 of/erythromycin	structurally identified relevant residue	MM-CoA/SNAC-derivatives	[63,64]
	AT from module 6/erythromycin	saturation mutagenesis of relevant motifs	MM-CoA/non-natural alkynyl-modified unit	[65]
AT-cross complementation	AT6null (inactivated AT in module 6)/erythromycin	*S. coelicolor* malonyl-CoA: ACP transacylase	MM-CoA/M-CoA	[66]
	AT6null (inactivated AT in module 6)/erythromycin	AT (BryP-AT1)/bryostatin	MM-CoA/M-CoA	[67]
	AT6null (inactivated AT in module 6)/erythromycin	AT (BryP-AT2)/bryostatin	MM-CoA/MM-CoA	[67]

## Abbreviations

ACC	acetyl-CoA carboxylase
ACP	acyl carrier protein
AT	acyltransferase
CCR	crotonyl-CoA carboxylase/reductase
CCRC	crotonyl-CoA reductase/carboxylase
CoA	coenzyme A
CRISPR-Cas9	clustered regularly interspersed palindromic repeats; CRISPR associated (Cas) protein (Cas9)
Cryo-EM	cryo-electron microscopy
KS	ketosynthase
DD	docking domains
DEBS	6-deoxyerythromycin B synthase
DH	dehydrogenase
ECR	enoyl-CoA carboxylase/reductase
ER	enoylreductase
KR	ketoreductase
NRPS	nonribosomal peptide synthetase
PCC	propionyl-CoA carboxylase
PKS	polyketide synthase
PPTase	4-phosphopantetheinyl transferase
Red/ET	homologous recombination system based on the Red operon of lambda phage or RecE/RecT from Rac phage
SNAC	*N*-acetylcysteamine
TAR	transformation-associated recombination
TE	thioesterase
USER	uracil-excision based cloning
YCC	acyl-CoA carboxylase
